# No significant histological or ultrastructural tendinosis changes in the hamstring tendon in patients with mild to moderate osteoarthritis of the knee?

**DOI:** 10.1007/s00167-020-06066-6

**Published:** 2020-06-05

**Authors:** Mustafa Ibrahim, Khaled Meknas, Sonja E. Steigen, Randi Olsen, Ninni Sernert, Lars Ejerhed, Jüri-Toomas Kartus

**Affiliations:** 1 Department of Orthopedics, NU Hospital Group, Trollhättan, Sweden; 2 Institution of Clinical Science, Sahlgrenska Academy, Gothenburg, Sweden; 3 Department of Orthopedics, University Hospital North Norway, Tromsø, Norway; 4 Orthopedics Research Group, Institute of Clinical Medicine, The Arctic University of Norway, Tromsø, Norway; 5 Diagnostic Clinic-Clinical Pathology, University Hospital of Northern Norway, Tromsø, Norway; 6 Institute of Medical Biology, Faculty of Health Sciences, The Arctic University of Norway, Tromsø, Norway; 7 Advanced Microscopy Core Facility, Institute of Medical Biology, UIT-The Arctic University of Norway, Tromsø, Norway; 8 Department of Research and Development, NU Hospital Group, Trollhättan, Sweden

**Keywords:** Hamstring, Tendinosis, Osteoarthritis, Knee

## Abstract

**Purpose:**

To investigate the periarticular degenerative changes of the knee joint in association with osteoarthritis (OA). More tendinosis was expected to be found in the semitendinosus tendon in patients with knee OA than in patients without knee OA.

**Methods:**

Samples from 41 patients were included between January 2016 and October 2017. Twenty-one patients median age 53 (33–63) years with mild to moderate OA underwent high tibial osteotomy (HTO) and 20 patients median age 38 (31–57) years without OA underwent anterior cruciate ligament reconstruction (ACLR). Biopsies from the semitendinosus tendon were obtained at the time of surgery and examined histologically, morphologically and ultrastructurally using light and electron microscope.

**Results:**

The histological evaluation of the semitendinosus tendon revealed the presence of more hemosiderin in the ACLR group. No significant morphological or ultrastructural differences were shown between patients in the HTO and ACLR group.

**Conclusion:**

Patients with mild and moderate medial compartment knee OA displayed no more degenerative changes in their semitendinosus tendon than patients without OA, as seen in both the light and the electron microscope.

**Level of evidence:**

III.

## Introduction

Osteoarthritis (OA) of the knee involves the degeneration of the intraarticular structures of the joint, such as cartilage degradation, subchondral bone sclerosis, synovitis with joint effusion and osteophyte and cyst formation [5]. These changes are well studied. Periarticular structures like muscles, tendons, ligaments are also often afflicted by OA. However, the presence of periarticular degeneration is not as outlined.

Changes in the periarticular tissues around the knee and other joints have been shown in some studies. Rutherford et al. investigated lower extremity electromyograms during walking and found that lower extremity neuromuscular function was altered with the presence and severity of knee OA [21]. The amount of quadriceps intramuscular fat is greater in patients with knee OA and it is also related to the severity of OA [13]. It has been reported that quadriceps weakness is associated with knee OA and thigh muscle strength is able to predict the risk of future knee replacement [4, 6, 18, 23].

There are few studies of periarticular tendon changes in association with OA of the knee. Yoon et al. have reported that an MRI signal alteration and abnormal thickening of the distal semimembranosus tendon are strongly associated with OA and medial collateral ligament thickening [28]. Meknas et al. showed that the internal obturator tendon in patients with OA of the hip had a more degenerative appearance compared with those without OA [15].

The corresponding finding has been reported in the shoulder by Ibrahim et al. [10].

The acquisition of more knowledge of periarticular tendon degeneration in association with OA is interesting, as early therapy targeted towards tendinosis might favorably alter the development and symptoms of OA [22].

The aim of the present study was to investigate the degenerative changes in the semitendinosus tendon in patients with mild to moderate knee OA and to compare them with tendons from patients with knee instability but without OA.

The hypothesis of the study was that more degenerative findings would be present in the semitendinosus in patients with knee OA than in patients without knee OA.

## Materials and methods

All patients gave their written consent and the Ethical Committee at the University of Gothenburg approved the study protocol (Dnr 381/15). A total of 41 consecutive patients participated in the study and underwent surgery between January 2016 and October 2017. With the aim of minimizing the inherent age discrepancy between the two groups, relatively young patients were selected for HTO (*n* = 21) and relatively old patients for ACLR (*n* = 20), (Table 1). During the study period, 24 patients in the HTO group and 130 patients in the ACLR group were scanned for eligibility.

**Table 1:** Age and gender of patients in the study groups

	HTO	ACLR
*n*	21	20
Age mean (SD)	50.7 (7.94)	41.9 (8.00)
Age median (range)	53 (33–63)	38 (31–57)
*P *value	**0.001**	
Female	11	12
Male	10	8
*P *value	n.s	

Significant value in bold

*n.s.* not significant, *n* number of patients, *SD* standard deviation

The inclusion criteria were primary medial compartment Ahlbäck grade 1–3 OA [1] of the knee or an unstable knee joint as a result of ACL rupture. The exclusion criteria were previous knee fracture (in both groups), arthritis with a genesis other than OA (in the OA group) and age over 65 years (in both groups). Furthermore, for the patients in the ACLR group, multi-ligament injuries and more than a grade II local chondral lesion according to the Outerbridge classification [17]. No patient in ACLR group displayed radiographic OA changes before surgery.

The material in this case control study consisted of samples from the semitendinosus tendon, obtained in an open fashion at the time of anterior cruciate ligament reconstruction (ACLR) or high tibial osteotomy (HTO). The semitendinosus tendon was chosen because it was easily accessible during both ACLR and HTO. Four samples were obtained from the semitendinosus tendon in each patient at the index operation. Each biopsy was about 0.5 × 0.5 cm large and was obtained 4 cm proximal to the tendon insertion on the tibia.

### Histological analysis

The samples for light microscopy were fixed in 4% formalin, embedded in paraffin blocks and sectioned at 4 μm. The sections were stained with hematoxylin–eosin (HE) to evaluate the fiber structure, cellularity and vascularity. Alcian blue/periodic acid Schiff (AB/PAS) was used to detect sour/neutral mucins for glycosaminoglycans (GAGs). Elastin staining was performed, staining collagen fibers red for easier detection. Furthermore, Perl’s, van Gieson and van Kossa stains were performed to identify hemosiderin, collagen and calcium deposits respectively. All the stainings were performed automatically (BenchMark Special Stains, Tucson, USA). The fiber structure, cellularity and vascularity and the presence of GAGs were classified according to a semi-quantitative scoring system (Table 2) [12]. It consists of four different elements, such as the fiber structure, cellularity, vascularity and GAGs. Each element can obtain between 0 and 3 points. This procedure and evaluation system have been performed in multiple previous studies [3, 8, 15, 25, 26].

**Table 2:** Evaluation of biopsy samples with a semi-quantitative four-point scoring system

	Grade 0	Grade 1	Grade 2	Grade 3
Fiber structure	Straight, parallel, packed fibers, with slight waviness	Slight separation of fibers, increased waviness	Separation of fibers, deterioration of fibers	Complete loss of fiber structure and hyalinization
Cellularity	< 100 cells/high-power field (HPF)	100–199 cells/HPF	200–299 cells/HPF	> 300 cells/HPF
Vascularity	Vessels running parallel to the collagen fiber bundles in the septa	Slight increase in vessels, including transverse vessels in the tendon tissue	Moderate increase in vessels within the tendon tissue	Markedly increased vascularity with clusters of vessels
Glycosaminoglycans	No alcianophilia	Slight alcianophilia between the collagen fibers	Moderate increase in alcianophilia	Markedly increased alcianophilia forming blue lakes

Subsequently, the total degeneration score (TDS) was calculated. The TDS can result in values between 0 (no degeneration at all) and 12 points (extremely high degeneration). The TDS is similar to a scoring concept previously described by Movin et al. [16] and used in a biopsy analysis of the Achilles tendon. The score has also undergone satisfactory intra-observer reliability testing [16].

The staining for hemosiderin and calcium deposits was dichotomously classified as positive/negative. The amount of scar tissue in the sample was estimated as a percentage of the field of view.

The histologic evaluations of two samples from each patient were performed by one independent pathologist (S.E.S.) with extensive experience. The pathologist was blinded to the group of specimen.

### Ultrastructural analysis

The ultrastructure was assessed using transmission electron microscopy (TEM) analysis and the specimens were fixed in 8% formaldehyde in Hepes buffer. The biopsies were cut into small cubes and half the material was immersion-fixed in McDowell’s fixative for electron microscopic studies [14]. After primary fixation, the tissue was washed with Sorensen’s phosphate buffer, post-fixated in 1% aqueous OsO_4_, washed and “en-bloc” stained with 2% uranyl acetate, dehydrated in a graded series of ethanol, embedded in an Epon substitute (AGAR: AGAR 100, MNA, DDSA) and DNP-30 with propylene oxide as a transitional solvent, according to standard procedures. Semithin and ultrathin sections were cut using a Leica Ultracut S (Vienna, Austria) on glass or diamante knives (Diatome, Biel, Switzerland). Ultrathin sections were mounted on formvar-coated 100 mesh copper grids and contrasted with 5% uranyl acetate, followed by Reynold’s lead citrate [20]. Micrographs were obtained using a Jeol JEM 1010 (Tokyo, Japan) with a Morada camera system (Olympus Soft Imaging Systems, Münster, Germany). For sampling, two blocks from each patient were sectioned and mounted on carbon-coated formvar films on copper grids. Micrographs for measuring the fibril diameters were obtained at random, from one to three groups of cross-sections from each block. The diameter of a minimum of 100 fibrils was measured using the Soft Imaging System (Olympus, Münster, Germany) at a magnification of 50,000. The relative fibril diameter distribution was calculated in percent. The diameters were grouped in six size classes (0–30, 31–60, 61–90, 91–120, 121–150 and > 150 nm). The accuracy of the measurements was 1/100th of an nm, but, in the results, an accuracy of 1/10th of an nm was chosen. This method has been used in a previous publication [10, 15]. The morphology of the extracellular matrix (ECM) was evaluated and dichotomously classified as homogeneous or irregular at a magnification of 2000.

The micrographs were evaluated by one independent technician (R.O.) with extensive experience of using the TEM. The technician was blinded to the group of specimen.

Two samples were scanned, but only the one with the best images was evaluated.

### Statistical analysis

Median (range) and mean (SD) values are presented for the TEM. For the histologic findings, a stratified distribution is presented. The unpaired *t *test and the Mann–Whitney *U* test were used for comparisons of the fibril diameters and the histologic findings respectively between the study groups. Since it has been shown that the distribution of fibril diameters in tendinopathic tendons exhibit a shift towards smaller diameters [19], the power analysis was based on the assumption that it would be meaningful to detect a difference of 5 nm in fibril diameter between the study groups. If the SD were as large as 40 nm, just over 1000 fibrils would need to be measured to reach a power of 80%. To increase the power of the study the comparison of the fibril diameter was based on almost 6000 fibrils, 2680 in the ACLR group and 3133 in the HTO group.

## Results

**Fig. 1:**
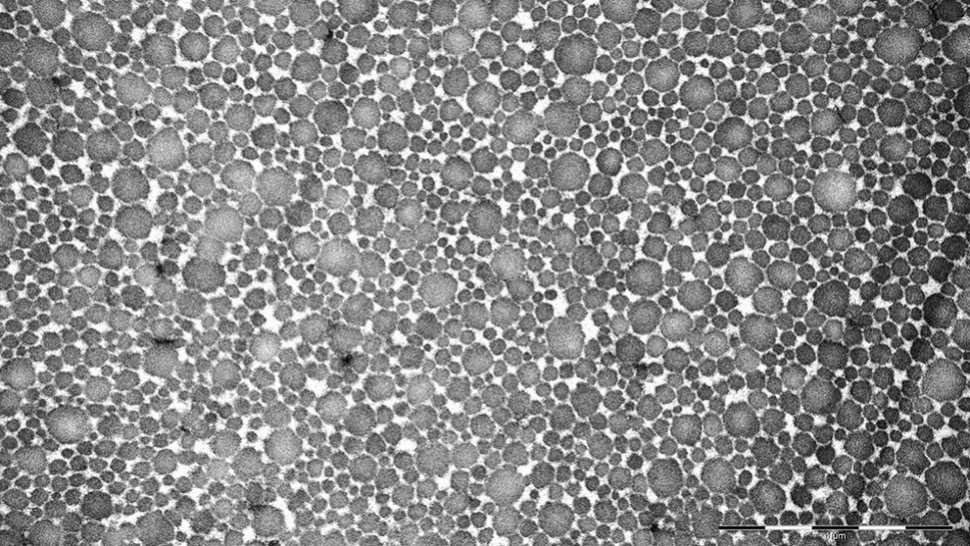
Semitendinosus tendon fibril diameters from a patient in the ACLR group, as seen in the electron microscope. Dominantly small and medium fibril diameters in a patient from the ACLR group. Original magnification ×50,000

**Fig. 2:**
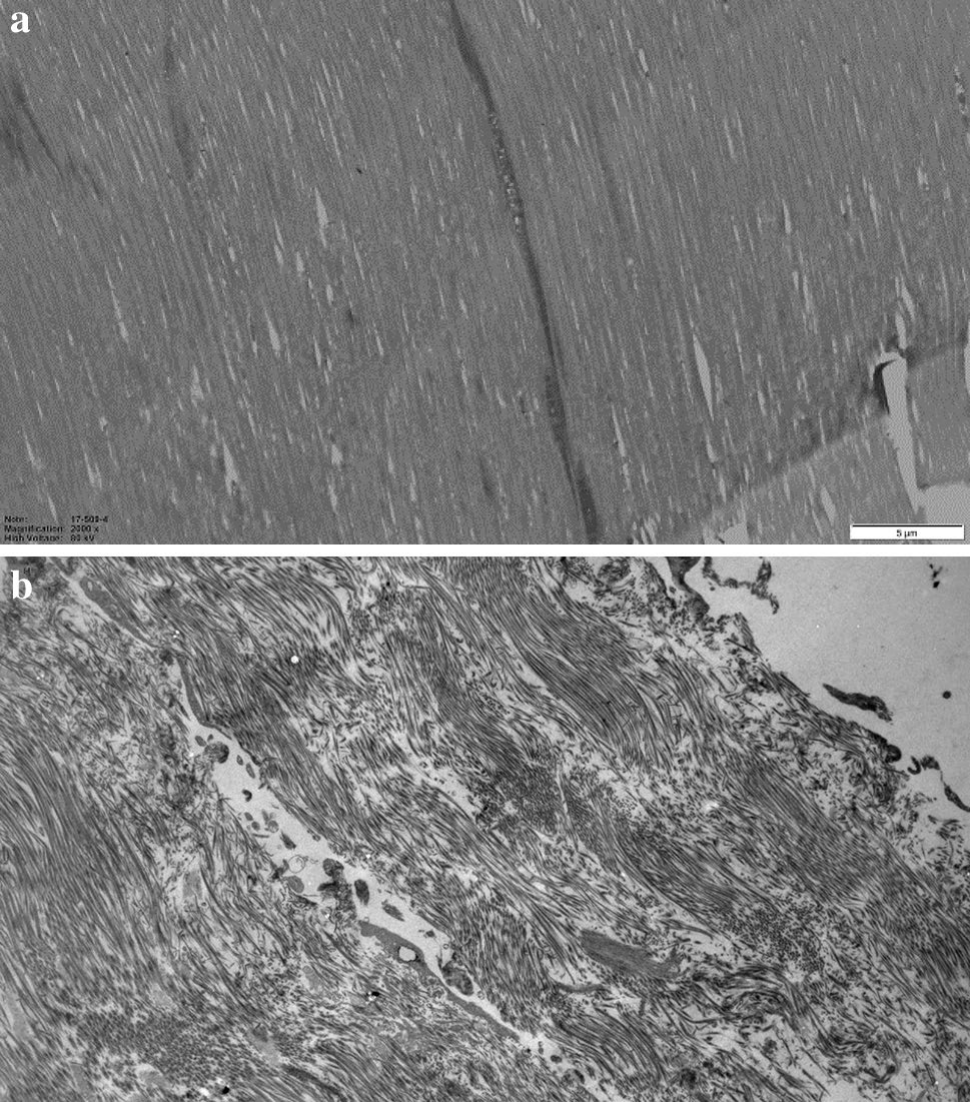
**a** Semitendinosus tendon morphology from a patient in the ACLR group. Homogeneous ECM with collagen fibrils running in the same direction. Original magnification ×2000. **b** Semitendinosus tendon morphology from a patient in the HTO group. Collagen fibrils oriented in different directions and an irregular ECM. Original magnification ×2000

**Fig. 3:**
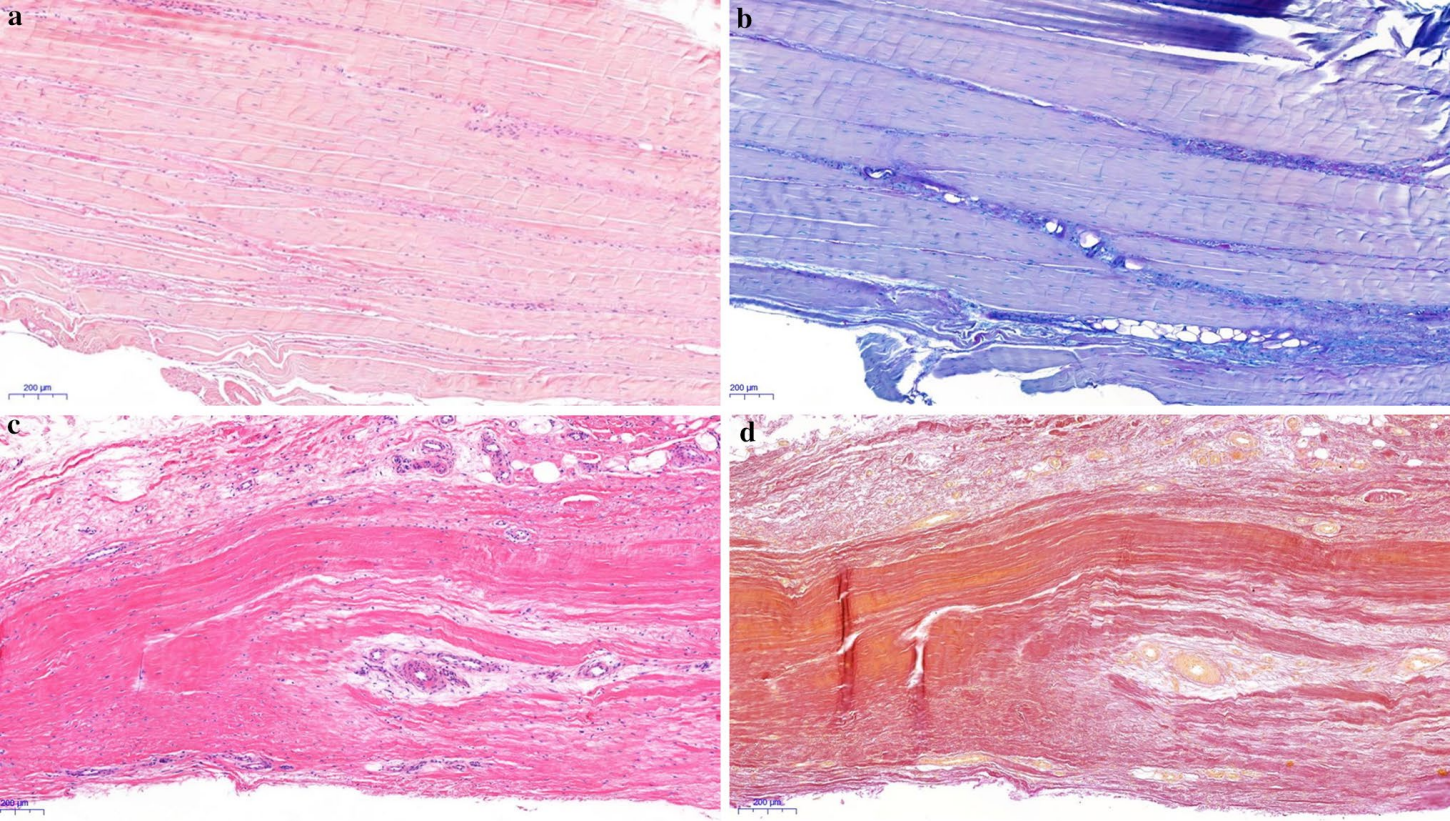
**a** Semitendinosus tendon from a patient in the ACLR group without associated OA, stained with hematoxylin–eosin. The fiber structure shows some increase in waviness. Original magnification ×50. **b** Semitendinosus tendon from a patient in the ACLR group without associated OA, stained with AB/PAS. Slight to moderate increase in alcianophilia (light blue stain) between the fibrous connective tissue. Original magnification ×50. **c** Semitendinosus tendon from a patient in the HTO group with associated OA, stained with hematoxylin–eosin. Separation and deterioration of the fibers. An increase in vessels within the tendon tissue. Original magnification ×50. **d** Semitendinosus tendon from a patient in the HTO group with associated OA, stained with elastin van Gieson. No convincing evidence of scar tissue. Original magnification ×50

Thirteen patients had mild OA (Ahlbäck grade 1), six patients had moderate OA (Ahlbäck grade II) and two patients had moderate to severe OA (Ahlbäck grade III) in the HTO group. The period of time between ACL injury and ACLR was 7 (2–28) months. The ultrastructural, (Tables 3, 4, Fig. 1), morphological (Table 5, Fig. 2a, b) and histological evaluation (Table 6, Fig. 3a–d) of the semitendinosus tendon all failed to reveal any significant differences between the patients in the HTO group and ACLR group, with the exception of the presence of more hemosiderin deposition in the ACLR group.

**Table 3:** Fibril diameter, electron microscopy

Fibril diameter (nm)	ACLR	HTO	*P *value
*n*	2680	3133	
Mean (SD)	80.0 (41.3)	78.7 (38.9)	n.s
Median (range)	66.7 (19.7–287.9)	65.1 (18.7–282.5)	

*n.s.* not significant, *n* number

**Table 4:** Relative distribution of fibril diameters

Fibril diameter (nm)	1–30	31–60	61–90	91–120	121–150	> 150
ACLR	2.6%	40.1%	34.6%	12.4%	6.6%	3.7%
HTO	0.5%	41.0%	35.4%	12.6%	6.3%	4.2%
*P *value	n.s					

*n.s.* not significant

**Table 5:** Extracellular morphology, electron microscopy

	ACLR	HTO	Total
Irregular	5	8	13
Homogeneous	15	13	28
Total	20	21	41
*P *value	n.s		

*n.s.* not significant

**Table 6:** Histology

	ACLR (*N* = 40)	HTO (*N* = 42)	*P *value
Fiber structure (%)			
0	5 (12.5)	8 (19)	n.s
1	19 (47.5)	20 (48)	
2	11 (27.5)	10 (24)	
3	5 (12.5)	4 (9)	
Cellularity (%)			
0	42 (100)	42 (100)	n.s
1	–	–	
2	–	–	
3	–	–	
Vascularity (%)			
0	19 (47.5)	23 (55)	n.s
1	20 (50)	17 (40)	
2	1 (2.5)	–	
3	–	2 (5)	
Glycosaminoglycans (%)			
0	35 (87.5)	36 (86)	n.s
1	4 (10)	4 (9)	
2	1 (2.5)	2 (5)	
3	–	–	
Calcium deposits (%)			
Negative	39 (97.5)	42 (100)	n.s
Positive	1 (2.5)	–	
Hemosiderin			
Negative	36 (90)	42 (100)	**0.037**
Positive	4 (10)	–	
Scar tissue (%)			
0	32 (80)	32 (76)	n.s
5	1 (2.5)	2 (5)	
10	1 (2.5)	3 (7)	
20	3 (7.5)	4 (10)	
30	2 (5)	1 (2)	
50	1 (2.5)	–	

Significant value in bold

*n.s.* not significant

The TDS revealed no significant differences between the groups (Table 7).

**Table 7:** Total degeneration score

Sum (0–12)	ACLR	HTO	*P* value
0 (%)	3 (7.5)	2 (4.8)	
1 (%)	9 (22.5)	17 (40.5)	
2 (%)	16 (40.0)	8 (19.0)	
3 (%)	5 (12.5)	12 (28.6)	
4 (%)	7 (17.5)	1 (2.4)	
5 (%)	–	2 (4.8)	
Mean (SD)	2.1 (1.17)	2.0 (1.20)	n.s
Median (range)	2.0 (0–4)	2.0 (0–5)	

*n.s.* not significant

## Discussion

The most important finding of the present study was that no significant histological, morphological or ultrastructural differences were found in the semitendinosus tendon between patients with mild to moderate knee OA compared with the control patients without OA.

This differs from the results of other similar studies in this field, as ultrastructural degenerative changes have been shown in periarticular tendons in both the shoulder and hip in association with OA [10, 15]. It is important to point out here that the patients in the two above-mentioned studies had severe OA changes that required surgical measures with shoulder or hip arthroplasty. The study population in the present study, on the other hand, had mostly mild to moderate OA changes.

In the present study, more than half the patients in the HTO group had mild OA. This might influence the results, as less tendon degeneration has to be expected with less radiological OA.

The patients in ACLR group were probably more physically active than the patients in the HTO group. It is known that young, active patients are more prone to traumatic knee injuries, including tendon injuries, and the ACLR group might therefore not have been the perfect control group [7].

The ultrastructural and histological evaluations, with the exception of the presence of hemosiderin, did not reveal any difference between the study groups. Possible explanations of why no significant differences were found in terms of histology might be the small study size, as the power analysis was based on the fibril diameter. Furthermore, the semitendinosus tendon degeneration might have been worse if patients with more advanced knee OA had been included. The hamstring tendons also play a role in reducing the anterior tibial translation after ACL rupture, which might lead to more tendon degeneration in the ACLR group than expected during the time period between ACL injury and reconstruction [11, 27]. The presence of hemosiderin, which is an indicator of a prior injury, was significantly higher in the ACLR group. This finding is interesting, as the semitendinosus is the second most frequently injured muscle in the hamstring in athletes [7]. However, the possibility that it could be a statistical artefact caused by multiple comparisons must be considered.

Some of the histological features in tendinopathy are usually a disorganization of the collagen fibers, an increase in the number of vessels and sensory nerves, a haphazardly arranged proliferation of smaller fibers and both hypocellularity (due to cell death) and hypercellularity (due to a fibroblast reaction) [22]. At the biochemical level, the cells in painful tendons produce increased levels of GAGs compared with normal tendon cells [22].

To the authors’ knowledge, this is a rare study in which the histological, morphological and ultrastructural changes in a periarticular tendon in the knee in patients with knee OA have been studied and compared with the same tendon from patients without OA.

The strengths of the study include the fact that the biopsies were obtained from living humans and that efforts were made to reduce the inherent age difference between the study groups. The age difference was inevitable, but the fact that age has not been shown to be a source of bias when comparing fibril diameters in both the hip and the shoulder in previous publications must be taken into account [8, 15]. Furthermore, Gagliano et al. have shown that the morphological and molecular characteristics of the hamstrings tendon were not influenced by age [9]. The limitations of the study include the fact that it might be under-powered, as well as the lack of preoperative activity level and symptom assessments. Furthermore, a non-optimal group of patients undergoing ACLR served as controls, as it was ethically impossible to obtain semitendinosus tendon biopsies from healthy age-matched individuals. Lastly, the hamstring tendon might not be the most appropriate tendon in which to study tendon degeneration due to its ability to regenerate itself, as it does after been harvested during ACLR [2, 24]. It might have been more appropriate to obtain biopsies from the quadriceps or patellar tendons, as these tendons lack the ability to regenerate themselves.

The findings in the present study and the previously mentioned studies raise the question of whether degenerative changes in the semitendinosus tendon might only occur in association with severe OA. However, to answer this question further studies are needed. Furthermore, the present study indicates that therapy specifically targeted towards tendinosis may not be as important as general strengthening exercises in early stages of OA.

## Conclusion

Patients with mild and moderate medial compartment knee OA displayed no more degenerative changes in their semitendinosus tendon than patients without OA, as seen in both the light and the electron microscope.

## Abbreviations

OA: Osteoarthritis
HTO: High tibial osteotomy
ACLR: Anterior cruciate ligament reconstruction
HE: Hematoxylin–eosin
AB/PAS: Alcian blue/periodic acid Schiff
GAGs: Glycosaminoglycans
TDS: Total degeneration score
TEM: Transmission electron microscopy
ECM: Extracellular matrix
